# Large cell neuroendocrine carcinoma of the lung presenting as pseudoachalasia: a case report

**DOI:** 10.1186/s13256-015-0514-y

**Published:** 2015-03-12

**Authors:** Ahmad Anaizi, Amna Rizvi-Toner, Jessica Valestin, Ron Schey

**Affiliations:** 1 Division of Gastroenterology and Hepatology, Department of Internal Medicine, University of Iowa Carver College of Medicine, 200 Hawkins Drive, 52240 Iowa City, IA USA; 2 Department of Medicine, Section of Gastroenterology, Temple University School of Medicine, 3401 n Broad street, 19140 Philadelphia, PA USA

## Abstract

**Introduction:**

Pseudoachalasia is a rare disease that accounts for only a small percentage of patients with dysphagia. Neuroendocrine tumors are rare malignancies that most commonly originate within the gastrointestinal tract, with the next most common site being the lungs. Esophageal neuroendocrine tumors are the least common site within the gastrointestinal tract. Pseudoachalasia can be secondary to a malignant process within the body. Its typical characteristic in elderly patients is a short duration of symptoms with substantial weight loss.

**Case presentation:**

A 68-year-old woman presented with worsening dysphagia that had started six months after the resection of a large cell neuroendocrine carcinoma of the lung in 2011. An extensive work-up in 2012, including esophagogastroduodenoscopy, chest computed tomography and positron emission tomography, was unremarkable. Esophageal manometries revealed findings characteristic of achalasia. A repeat esophagogastroduodenoscopy in January of 2014 revealed a nearly circumferential ulcerated, fungating mass in her distal esophagus. Biopsy results confirming a recurrence of her large cell neuroendocrine carcinoma.

**Conclusion:**

We report a case of pseudoachalasia due to metastatic large cell neuroendocrine carcinoma of the lung. Our patient had an exceptionally prolonged duration of symptoms preceding the local esophageal recurrence, which was eventually revealed via endoscopy.

## Introduction

Neuroendocrine neoplasms may arise in a number of organs and come from cells containing vasoactive substances within secretory granules located in the cytoplasm [1]. Neuroendocrine tumors can be classified further into large cell neuroendocrine carcinoma (LCNEC), small cell lung carcinoma (SCLC), and typical and atypical carcinoid tumors [2]. The majority of these tumors arise from the gastrointestinal tract and are known as gastropancreatic neuroendocrine tumors. The next most common site for neuroendocrine tumors is in the lungs. LCNEC was not introduced as a distinct entity until 1991; these tumors have a similarly grim prognosis to SCLC but have a cell size of at least three times that of SCLC, as well as an organoid growth pattern [3].

Achalasia is a condition in which there is a loss of inhibitory neurons of the myenteric plexus within the wall of the esophagus. In pseudoachalasia, the patient presents with clinical and manometric findings consistent with achalasia, but the symptoms are caused by a secondary organic entity. Pseudoachalasia needs to be excluded in older patients (>60 years) who have a short duration of symptoms (<one year) and substantial weight loss [4]_._ Malignancy-associated pseudoachalasia can occur via one of three ways: the cancer can be located at the gastroesophageal junction and inhibit swallowing by mass effect; esophageal neuronal invasion by the malignancy can lead to a disruption of neuronal transmission, resulting in dysmotility of the esophagus and consequent dysphagia; or a pseudoachalasia can be associated with a paraneoplastic process [5]. The latter process is secondary to an autoimmune reaction whereby host T cells recognize antigens expressed by the tumor and cross-react with various parts of the central and peripheral immune system. The most common autoantibody seen with this phenomenon is type 1 antineuronal nuclear autoantibody, also referred to as Anti-Hu antibody [6].

We present the case of a patient with pseudoachalsia due to metastatic LCNEC, who had a negative extensive workup preceding luminal manifestation for over two years.

## Case presentation

Our patient was a 68-year-old woman with chronic obstructive pulmonary disease. She had no history of gastroesophageal reflux disease or esophageal disorders but was found to have a progressive solitary pulmonary nodule (1×1.3cm). A right lower wedge resection was performed in December 2011 and the University of Iowa pathology department identified the tumor as a LCNEC.

**Figure 1:**
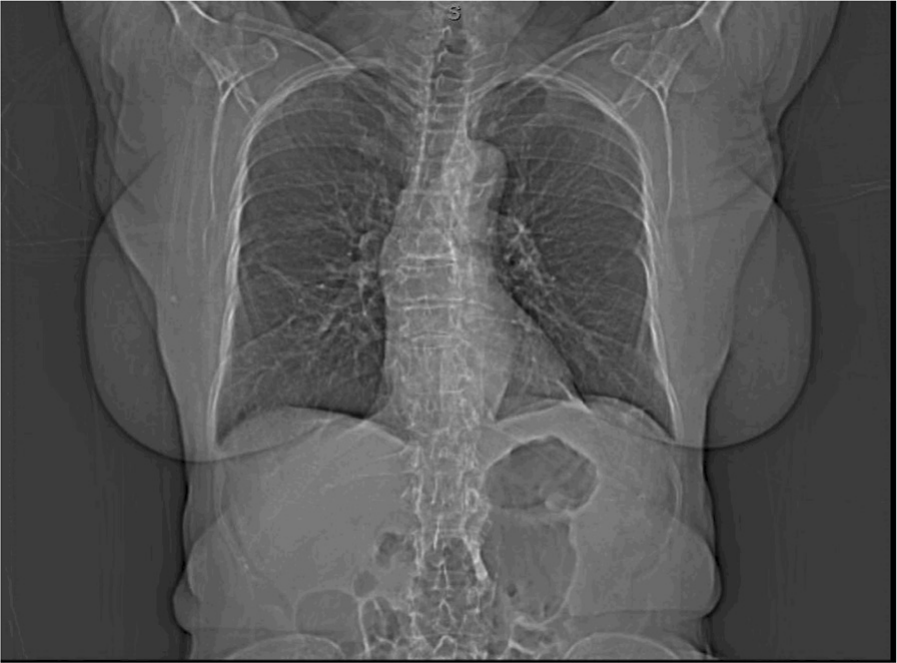
**Coronal view of a computed tomography scan of the chest.**

Approximately six months after her operation, our patient started having slowly progressing dysphagia for both solids and liquids. A positron emission tomography (PET) scan performed in 2012, one year post surgery, was unremarkable for recurrence or metastasis. Repeat computed tomography (CT) in June 2013 (18 months post surgery) did not show any recurrence (Figure 1). Our patient had an esophagogastroduodenoscopy (EGD) and barium swallow that revealed no stricture of her esophagus but failure of primary and secondary peristaltic waves and reflux. In July 2013, esophageal manometry found failure of the lower esophageal sphincter to relax, with a residual pressure of 32mmHg. Our patient was treated with calcium channel blockers with partial relief.

**Figure 2:**
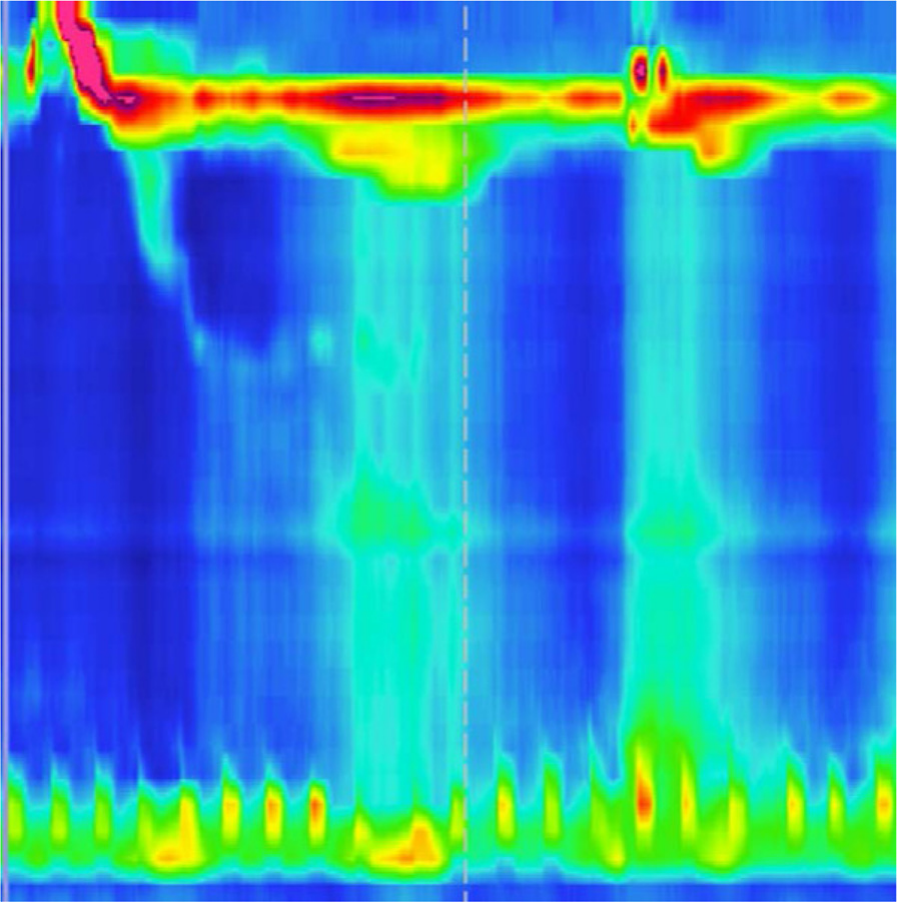
**Esophageal manometry demonstrating the patients type 1 achalasia.**

Thus far, up to 18 months post resection, our patient had been able to maintain her weight and her basic laboratory parameters, including hemoglobin, albumin and cholesterol levels. However, in January 2014, she reported worsening dysphagia and weight loss of 10lbs over a period of one month. She was referred to our center for further treatment of her achalasia. A repeat esophageal manometry showed an elevated lower esophageal sphincter pressure of 50mmHg as well non-propagative, non-peristaltic contractions throughout her esophageal musculature (Figure 2). A repeat EGD with an intention to perform therapeutic pneumatic dilation showed a nearly circumferential ulcerated, fungating mass from approximately 36cm that extended to her gastroesophageal junction at 39cm. Biopsies revealed a LCNEC with similar features to the primary tumor resected in 2011, and was concluded to be a metastasis. Subsequently, a repeat CT scan revealed progressive diffuse metastatic disease. Our patient underwent two courses of chemotherapy but died from the disease after six months.

## Conclusion

Pseudoachalasia is rare, and approximately 5% of patients with manometric findings of achalasia have a malignant disorder. The primary malignancies are mainly of the esophagus or gastroesophageal junction (over 50%), followed by metastases (mainly to the lung and breast) of paraneoplastic syndromes in the context of small-cell carcinoma, bronchial carcinoma, gastric carcinoma and pleural mesothelioma [7].

We report a case of pseudoachalasia due to metastatic LCNEC of the lung in a 68 year-old woman, with mild onset of symptoms for two years prior to substantial weight loss. Over one year after her surgery, our patient had normal findings on an EGD, chest CT and PET scan. Her manometric and radiological findings were consistent with progressive achalasia. Although it might be difficult to diagnose in an early phase, it is very uncommon for a patient with metastatic pseudoachalasia to maintain weight and clinical stability for a period as long as over two years before establishment of luminal findings.

We would like to suggest here that in an older patient newly diagnosed with achalasia, regardless of normal CT and PET scan results over one year after tumor resection, persistence or worsening of symptoms with extensive weight loss should indicate thorough and repeat work-up until a diagnosis of metastatic pseudoachalasia can be ruled out.

## Consent

Written informed consent was obtained from the patient for the publication of this report and any accompanying images.
